# The Use of Diatoms in the Synthesis of New 3D Micro-Nanostructured Composites (SiO_2_/CaCO_3_/C_org_/NdVO_4_NPs and SiO_2_/CaO/C_org_/NdVO_4_NPs) Exhibiting an Intense Anti-Stokes Photoluminescence

**DOI:** 10.3390/ma17020490

**Published:** 2024-01-19

**Authors:** Weronika Brzozowska, Izabela Wojtczak, Myroslav Sprynskyy

**Affiliations:** 1 Division of Surface Science, Faculty of Chemical Technology and Engineering, Bydgoszcz University of Science and Technology, 7 Kaliskiego Str., 85-796 Bydgoszcz, Poland; weronika.brzozowska@pbs.edu.pl; 2 Department of Environmental Chemistry and Bioanalytics, Faculty of Chemistry, Nicolaus Copernicus University in Toruń, 7 Gagarina Str., 87-100 Torun, Poland; izabelawojtczak1991@gmail.com

**Keywords:** anti-Stokes photoluminescence, diatom biomass, metabolic insertion, neodymium vanadate nanoparticles

## Abstract

New 3D micro-nanostructured composite materials have been synthesised. These materials comprise SiO_2_/CaCO_3_/C_org_/NdVO_4_NPs and SiO_2_/CaO/C_org_/NdVO_4_NPs, exhibiting strong upconversion luminescence. The synthesis was accomplished by metabolically doping diatom cells with neodymium and vanadium. Subsequently, the biomass of these doped diatoms was subjected to pyrolysis at 800 °C. The morphology, structure, and physicochemical properties of the doped diatom biomass as well as dried (SiO_2_/CaCO_3_/C_org_/NdVO_4_NPs) and pyrolysed (SiO_2_/CaO/C_org_/NdVO_4_NPs) samples were characterised using scanning electron microscopy (SEM), scanning transmission electron microscopy (STEM), energy dispersive X-ray spectroscopy (EDX), X-ray powder diffraction (XRD), thermal analysis (TG), and fluorescence spectroscopy (FS). Studies have shown that the surface of diatom shells is covered with trigonal prismatic nanocrystallites (nanoparticles) of NdVO_4_ with dimensions of 30–40 nm, forming the crystallite clusters in the form of single-layer irregular flakes. The synthesised composites produced intense anti-Stokes fluorescent emission in the visible region under xenon lamp excitation in the near-infrared (λ_ex_ = 800 nm) at room temperature in an ambient atmosphere. Such materials could be attractive for applications in solar spectrum conversion, optical sensing, biosensors, or photocatalysts.

## 1. Introduction

In recent years, rare earth elements (REEs) have garnered considerable attention. Despite their name, most REEs are not rare in terms of abundance, and the term ‘rare’ should be more concerned with the complex process of extracting them, as such elements tend to occur together in nature and are not easily separated from each other [1]. Although REEs are characterised as separate components, each element has a specific set of practical applications. Rare earth metals find applications in various fields, including modern civilian and military technologies, as well as in medicine, optics, nuclear technologies, telecommunications, and the aerospace industry [2,3]. In biology and medicine, REEs can have diverse applications. For example, gadolinium is used in magnetic resonance imaging (MRI) due to its ability to enhance image quality [4]. Other rare earth metal ions can be used as markers in biochemical and biomedical studies. Additionally, research is being conducted on the use of REEs in radiotherapy, where the radioactive isotopes of some REEs may be utilised to destroy cancer cells [5]. Unique optical properties of REEs, such as broad optical transparency, photoluminescence, and anti-Stokes photoluminescence, are mainly due to the diversity of transitions within the 4f n electron states [6]. The excitation efficiency 4f in REE ions can be increased by transferring the charge from the host material with a higher absorption coefficient. A good candidate for the attachment of trivalent lanthanide ions is the orthovanadate groups VO_4_^3−^ because in such a connection, there is an energy transfer to the emission lanthanide ions through charge transfer in the VO_4_^3−^ groups [6]. Therefore, for several reasons, orthovanadates of rare earth metals are one of the key classes of inorganic functional materials based on REEs. Orthovanadates exhibit unique optical properties, such as photoluminescence [7]. Thanks to these properties, they are used to produce optical materials and lasers [8]. Some orthovanadates have the ability for photocatalysis [9], used for breaking down pollutants in the environment, such as dyes and organic compounds, making them important in environmental protection. Orthovanadates can also demonstrate magnetic properties, making them useful in the fields of magnetism and materials engineering [10]. The rare earth orthovanadates are, therefore, one of the key classes of inorganic REE-based functional materials. Among rare earth vanadates, NdVO_4_ belongs to the zirconium structure with space group $D_{4h}^{19}$ [11]. Consequently, the lanthanide ion in this crystal has low symmetry, which promotes electrical dipole transformations, which results in higher radiation rate constants and decreased quenching processes. Due to its properties and potential benefits for industry and science, neodymium vanadate is one of the most extensively studied orthovanadates within the lanthanide orthovanadate group ABO_4_. These crystallise in a tetragonal structure formed of a slightly distorted tetrahedral VO_4_^3−^ ion and a rare earth ion Nd^3+^ between adjacent tetrahedrons. Each Nd^3+^ ion is dihedrally surrounded by eight oxygen ions [12]. 

**Figure 1:**
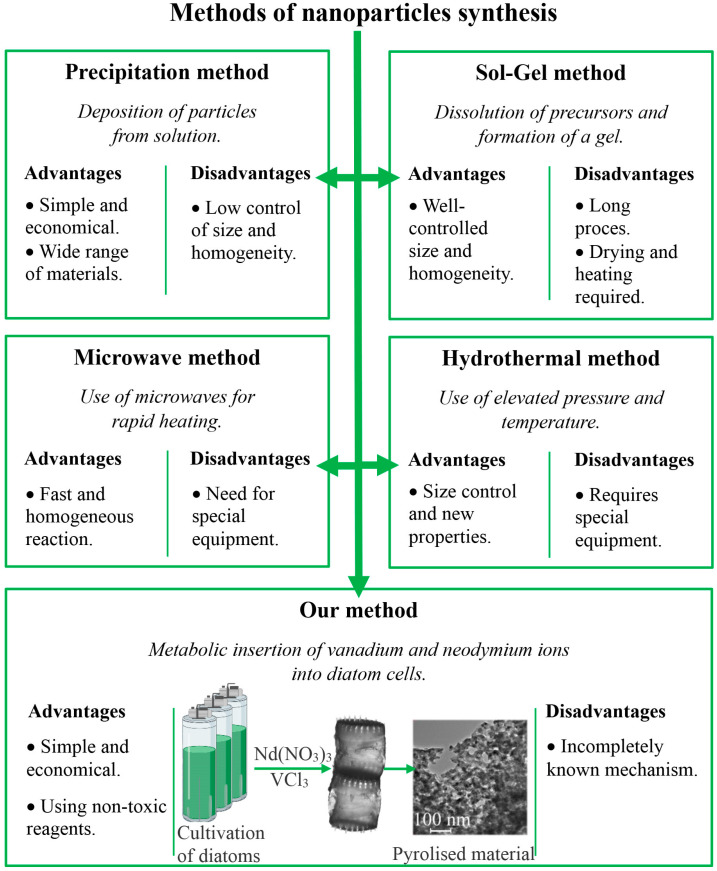
Comparison of synthesis methods of NdVO_4_ nanoparticles (advantages and disadvantages) with the method described in this paper.

There have been numerous studies of NdVO_4_-based optical materials [13,14,15]. Y-doped NdVO_4_, a well-known laser material, has an absorption coefficient at 808 nm that is five times higher than that of the Nd:Y_3_Al_5_O laser diode [14]. In addition, NdVO_4_ has been demonstrated to exhibit photocatalytic activity for the degradation of dyes and organic pollutants, which is comparable to or even higher than commercial TiO_2_ [13,16]. The neodymium vanadate in nanocrystalline forms, with their large specific surface areas and quantum size effects, offer properties not usually observed in bulk [2]. Consequently, lanthanide orthovanadates in nanocrystalline form exhibit properties that make them potential multiphoton photoluminescence materials for solar cells, photocatalysts, light-emitting diodes, biosensors, and contrast agents in bioimaging [17,18,19,20,21]. However, several methods have been developed to synthesise NdVO_4_ nanostructures (hydrothermal, microwave or sonochemical synthesis, co-precipitation, and metathesis reactions). Yuvaraj et al. described the synthesis of NdVO_4_ nanoparticles using the precipitation method. This method involves the simultaneous precipitation of neodymium and vanadium from a solution as a precipitate. The precipitate is then subjected to thermal treatment to obtain NdVO_4_ nanoparticles [11]. In the work by Monsef and co-authors, NdVO_4_ nanoparticles were synthesised using the sol-gel method, which involves dissolving neodymium and vanadium precursors in suitable solvents. A gel is then formed, dried, and heated to produce NdVO_4_NPs [22]. Mahapatra et al. reported the microwave-assisted synthesis of NdVO_4_ nanoparticles. This method uses microwaves to rapidly uniformise precursors, potentially leading to more homogeneous nanoparticles [13]. In the study by Wu and co-authors, the synthesis of NdVO_4_ nanoparticles was described using the hydrothermal method, which involves elevated pressure and temperature to synthesise nanoparticles in a stainless steel autoclave lined with Teflon in a digitally controlled temperature furnace [7]. All of these methods have specific disadvantages—the high treatment temperatures, long reaction times, expensive instrumentation, or the use of toxic solvents [12]. An environmentally friendly approach to synthesising NdVO_4_ nanoparticles using a natural template allows for conducting reactions under milder conditions (Figure 1). Biological synthesis processes based on biotemplates enable microorganisms as carriers to produce functional MNPs [23].

Microorganisms provide stability and material modification capabilities. The ordered assembly of MNPs based on microorganisms enhances the efficiency of functional materials and composite structures, imparting them with new properties [24]. Moving microorganisms inspire the design of micro/nanorobots for biomedical applications [25]. However, challenges related to scalability, efficiency, and uniformity in the synthesis process exist. Although these technologies are in their early stages, a deeper understanding of MNP synthesis and assembly mechanisms opens the door to advanced future applications. Considering the mentioned advantages of synthesising nanoparticles on natural carriers, an eco-friendly approach, and a wide range of potential applications, this study used diatoms of the *Pseudostaurosira trainorii* as a matrix in synthesising NdVO_4_ nanoparticles.

In this work, we present the study results of the biosynthesis of new 3D micro-nanostructured composite materials (SiO_2_/CaCO_3_/C_org_/NdVO_4_NPs and SiO_2_/CaO/C_org_/NdVO_4_NPs) with anti-Stokes photoluminescence using the metabolic doping of diatom cells by neodymium and vanadium during the process of diatoms cultivation. We describe a green method to obtain crystalline NdVO_4_ nanoparticles covering diatom cells in the cluster forms. 

## 2. Materials and Methods

The selected diatom strain was identified as *Opephora* sp. from the Collection of Baltic Algae Cultures of the Institute of Oceanography of the University of Gdańsk. However, according to a more detailed study of the frustule morphology using scanning electron microscopy techniques, this diatom species was defined as *Pseudostaurosira trainorii* [26]. The diatom species were cultured in 25 L photobioreactors at 20 °C, with Guillard f/2 medium (adjusted to the final pH of 8.4) and under 24 h light regime using two 1500 lux fluorescent lamps. The initial concentration of soluble silicon (Na_2_SiO_3_·5H_2_O) (Merck, Darmstadt, Germany) in the medium was 7 mg Si/L. The initial concentration of neodymium (Nd(NO_3_)_3_) (Merck, Darmstadt, Germany) in the medium was 10 mg Nd/L, and the vanadium (VCl_3_) (Merck, Darmstadt, Germany) concentration was 10 mg V/L. The experiment of diatom biomass cultivation lasted for 12 days. 

The mechanism for obtaining NdVO_4_ nanoparticles from precursors (Nd(NO_3_)_3_ and VCl_3_) is as follows:

(1) $$Nd(NO_{3}{)}_{3}\overset{H_{2}O}{↔}{Nd}^{3+}+3NO_{3}^{-}$$

(2) $${VCl}_{3}\overset{H_{2}O}{↔}V^{3+}+3{Cl}^{-}$$

(3) $${2V}^{3+}+O_{2}+{12OH}^{-}↔{2VO}_{4}^{3-}+{6H}_{2}O$$

(4) $${Nd}^{3+}+{VO}_{4}^{3-}\overset{diatoms}{\to }{NdVO}_{4}↓$$

The obtained diatom biomass doped with neodymium and vanadium ions was divided into two parts. One part of the biomass was dried at 70 °C, and the other was pyrolysed under a nitrogen atmosphere using a high-temperature furnace. Pyrolysis of the sample was carried out at 800 °C for 4 h. As a result, the dried diatom biomass doped with neodymium and vanadium will take the abbreviation SiO_2_/CaCO_3_/C_org_/NdVO_4_NPs, and the doped pyrolysed biomass will take the abbreviation SiO_2_/CaO/C_org_/NdVO_4_NPs.

The morphological features and elemental composition of the prepared composites were investigated using a scanning electron microscope (SEM, LEO 1430 VP, Electron Microscopy Ltd., Cambridge, UK) coupled to an Energy Dispersive X-ray detector (XFlash 4010, Bruker AXS, Bremen, Germany), STEM transmission imaging using a scanning electron microscope SEM/FIB Quanta 3D FEG (FEI Company, Hillsboro, OR, USA), and Transmission Electron Microscopy (TEM, FEI Tecnai F20 X-Twintool, FEIEurope, Frankfurt/Main, Germany). The mineral composition of the composites was characterised using X-ray powder diffraction (XRD) using a Philips X‘Pert Pro diffractometer (XRD, Malvern Panalytical Ltd., Malvern, UK) with Cu-Kα (γ = 0.1541 nm, 40 kV, 30 mA). Analysis data were collected with a step size of 0.01 over an angular range of 10–80 2θ. The thermal stability was investigated using a thermogravimetric method. A thermoanalyser SDT 650 from TA Instruments was used for the analysis. The analysis was conducted in a nitrogen atmosphere at a 10 °C/min heating rate. Analysis was carried out up to a temperature of 1000 °C. The UV-vis absorption spectrum of the sample on quartz slices was measured with a Jasco V-750 spectrophotometer under normal incident light in the range 250–850 nm. Hitachi F-2500 fluorescence spectrophotometer equipped with a xenon lamp was applied for the photoluminescence (PL) properties measured of the synthesised composites. PL spectra were recorded at excitation wavelengths of 800 nm, at room temperature 20 °C in ambient atmosphere. Measurements were carried out for solid samples placed in a special cell. The slits used were 2.5 nm, the voltage was 700 V, and the scanning speed was 60 nm/min. According to the instrument’s capabilities, the measurement range was from 250 to 700 nm.

The fluorescence quantum yield of pyrolysed biomass doped with NdVO_4_ in ethanol solution was determined using a relative method. This involved comparing the unknown quantum yield of the sample with the known quantum yield of the reference dye in a fluorescence spectrometer. The theoretical prerequisite for the relative method is that the sample and reference solution have identical absorption at the excitation wavelength and, therefore, absorb the same number of photons. The ratio of the quantum yields of the sample and reference can be easily calculated by taking the quotient of the integrated fluorescence spectra (IF = fluorescence band area) of the two solutions, which were recorded under identical conditions:

(5) $$n_{{fl}_{sample}}=n_{{fl}_{reference}}\times \frac{{IF}_{sample}}{{IF}_{reference}}$$

If different solvents are used for the sample and standard, the refractive indices of these solvents should be entered into Formula (5):

(6) $$n_{{fl}_{sample}}=n_{{fl}_{reference}}\times \frac{{IF}_{sample}}{{IF}_{reference}}\times \frac{n_{sample}^{2}}{n_{reference}^{2}}$$

To determine the fluorescence quantum yield of the SiO_2_/CaO/C_org_/NdVO_4_NPs sample, two solutions were prepared: the sample solution—SiO_2_/CaO/C_org_/NdVO_4_NPs solution in 96% ethanol (Merck, Darmstadt, Germany) and the standard solution—quinine bisulphate solution (Satna Cruz Biotechnology, Heidelberg, Germany) in 0.5 M sulphuric acid(VI) (Merck, Darmstadt, Germany). The fluorescence spectrum of the sample and standard was recorded using a Hitachi F-2500 xenon lamp fluorescence spectrophotometer. The excitation wavelength used was 305 nm, at which the sample and reference solutions had identical absorption (A = 0.12).

## 3. Results and Discussion

**Figure 2:**
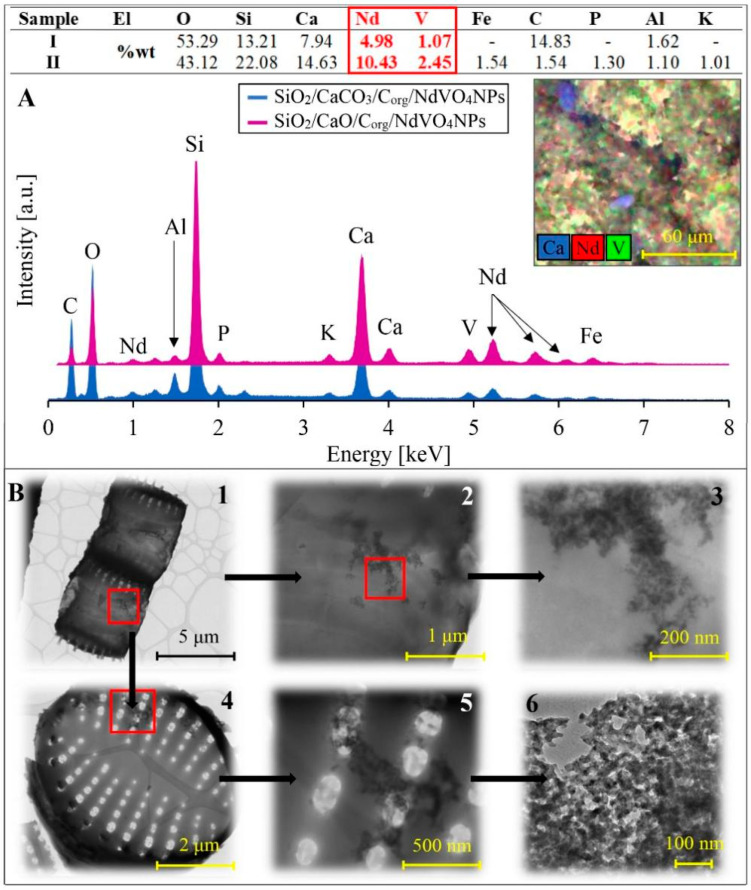
SEM-EDX spectra, the elemental composition of the obtained composites and a map of the distribution of doped elements in SiO_2_/CaCO_3_/C_org_/NdVO_4_NPs and SiO_2_/CaO/C_org_/NdVO_4_NPs (**A**). STEM images showing the morphology and structure of the pyrolysed diatom cells decorated with cluster forms of NdVO_4_ NPs (SiO_2_/CaO/C_org_/NdVO_4_NPs) at different magnifications (**B.1**–**B.5**), (**B.1**)—diatom cells in the form of colonial ribbons, (**B.4**)—the single diatom cell, (**B.6**)—TEM image of the single layer flake-likes cluster of the NdVO_4_ nanocrystallites.

**Figure 3:**
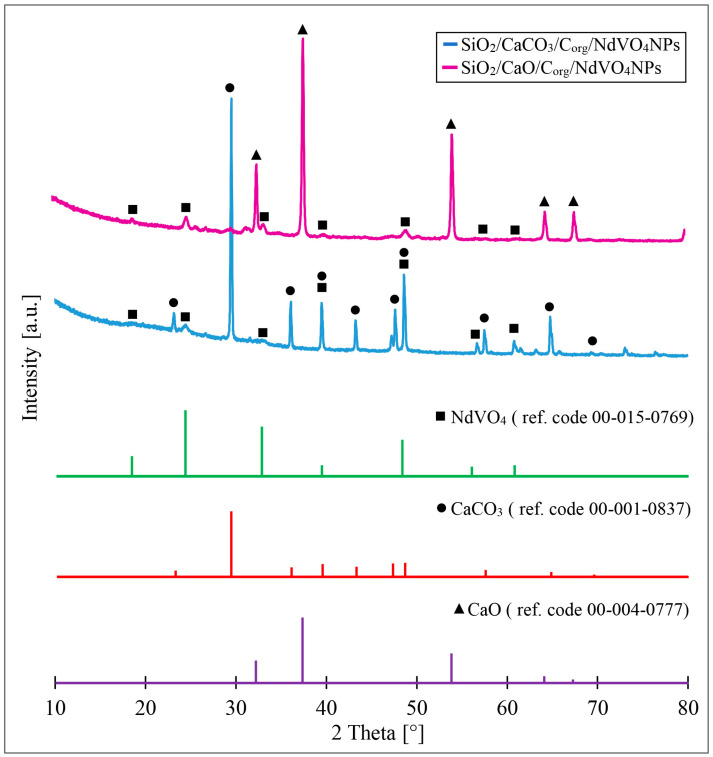
X-ray diffractograms of obtained composites and references; pink line—SiO_2_/CaO/C_org_/NdVO_4_NPs, blue line—SiO_2_/CaCO_3_/C_org_/NdVO_4_NPs, green line—NdVO_4_, red line—CaCO_3_, purple line—CaO.

**Figure 4:**
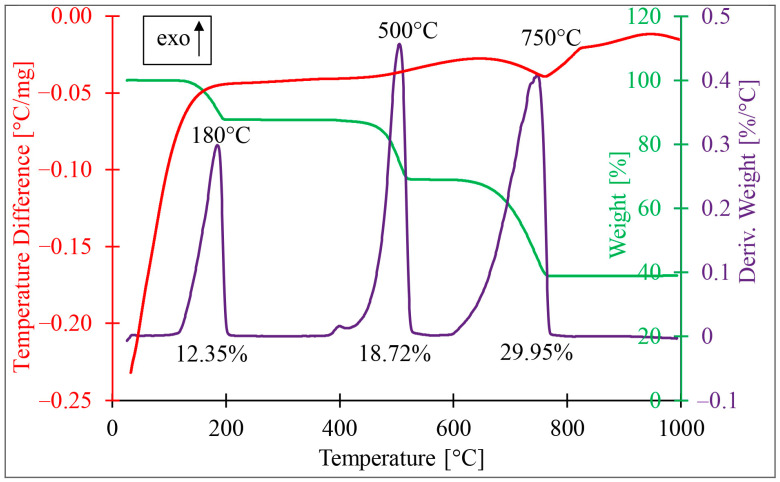
Phase transitions and thermal stability of the obtained composite. TG, thermogravimetric analysis (green line); DTG, thermogravimetric derivative analysis (purple line); and DSC, differential scanning calorimetry (red line).

Figure 2A presents the results of SEM-EDX spectral analysis. This analysis was performed on both dry and pyrolysed biomass doped with NdVO_4_. It revealed that the main components of the materials are oxygen, carbon, silicon, and calcium. A decrease in carbon content was observed in the pyrolysed sample. This contrasts with the sample that did not undergo thermal treatment. This suggests the presence of graphitised organic matter from diatom cells post-pyrolysis [27]. The high calcium content of the samples suggests the presence of calcium carbonates (in the sample without pyrolysis) or calcium oxide (in the sample after pyrolysis), as confirmed by XRD results (Figure 3). Both samples also had high neodymium and vanadium contents, amounting to 4.98% Nd and 1.07% V for SiO_2_/CaCO_3_/C_org_/NdVO_4_NPs and 10.43% Nd and 2.45% V for SiO_2_/CaO/C_org_/NdVO_4_NPs. The presence of neodymium and vanadium in both samples suggests that NdVO_4_ nanoparticles are formed at the beginning of the synthesis process, i.e., after adding neodymium and vanadium precursors to the culture medium. The mechanism of NdVO_4_NPs formation is presented using Equations (1)–(4) (in the Section 2). The formation of NdVO_4_ nanoparticles during the growth of diatom cells suggests that these single-cell microalgae participate in the process of NdVO_4_NPs formation. For this reason, there is a hypothesis that diatoms participate in forming NdVO_4_ nanoclusters, and the synthesis of NdVO_4_NPs itself occurs at the moment of formation of the silica shell of diatoms. However, the mechanism of the formation of the silica frustule itself is not fully understood, so we cannot explain in detail the process of forming nanocrystallite growths on the diatom shell. Taking into account the SEM-EDX data, the obtained composites can also be presented in the following forms, taking into account the percentage content of silicon, calcium, neodymium, and vanadium oxide compounds: 28%SiO_2_/20%CaCO_3_/15%C_org_/8%NdVO_4_NPs; 48%SiO_2_/21%CaO/2%C_org_/17%NdVO_4_NPs. Composite 28%SiO_2_/20%CaCO_3_/15%C_org_/8%NdVO_4_NPs also includes about 14% of bound water (see Figure 4), while composite 48%SiO_2_/21%CaO/2%C_org_/17%NdVO_4_NPs contains admixtures of iron, phosphorus, and potassium. The mapping performed for the obtained composites indicates an even distribution of neodymium and vanadium in these materials. 

The samples were also examined using a scanning transmission electron microscope to analyse the resulting nanoparticles’ morphology, arrangement, shape, and size. Figure 2B compares STEM images of diatomite composites containing NdVO_4_NPs without pyrolysis (B.1) and after pyrolysis (B.2). These images show the intricate structure of the diatoms. One can see the architecture of the entire diatom shell, the pore structure details, and the distribution, shape, and size of the resulting NdVO_4_ nanoparticles. Figure 2B.6 shows the TEM image of the NdVO_4_ crystallites (nanoparticles) with 30–40 nm dimensions forming the crystallite clusters in the thin film irregular flake forms. It is possible to see trigonal prismatic or square-plate forms of the NdVO_4_ nanocrystallites. The square-plate morphology is characteristic of nanoparticles of rare-earth element homologs, such as NdVO_4_ [28]. The size and location of these flakes on diatom frustules vary; however, their presence is characteristic of all composites, regardless of thermal treatment. We obtained similar coatings in our recent work on doping diatom biosilica with titanium ions [17]. However, the nature of the binding of the resulting flake to the surface of diatom frustules remains unexplained. This binding may be due to the combination of silanol groups and residuals of the proteins responsible for forming the silica shell (sylaphins) with the nanoparticle flake.

XRD patterns of dried and pyrolysed diatom biomass doped with NdVO_4_ are presented in Figure 3. The X-ray diffractograms obtained for SiO_2_/CaCO_3_/C_org_/NdVO_4_NPs showed distinct crystal peaks located at approximately 2θ = 18.80°, 24.51°, 33.03°, 39.65°, 48.66°, 56.29°, and 60.93°. The SiO_2_/CaO/C_org_/NdVO_4_NPs sample yielded peaks for 2θ values of 18.83°, 24.89°, 33.41°, 39.99°, 49.05°, 56.70°, and 61.31°. The values of the detected diffraction peaks are characteristic of neodymium vanadate (ref. code: 00-015-0769, NdVO_4_). The XRD spectrum of dried diatom biomass metabolically doped with NdVO_4_ (SiO_2_/CaCO_3_/C_org_/NdVO_4_NPs) also shows intense peaks located at 2θ = 23.17°, 29.48°, 36.14°, 39.55°, 43.31°, 47.33°, 48.73°, 57.59°, 64.87°, and 69.64°, which reflect peaks from calcite CaCO_3_ (ref. code: 00-001-0837). It proves that the mineralisation of CaCO_3_ has occurred at the diatom biomass’s drying stage. X-ray diffractogram obtained for SiO_2_/CaO/C_org_/NdVO_4_NPs composite shows clear peaks (2θ: 32.65°, 37.79°, 54.32°, 64.60°, and 67.85°) characteristic of CaO according to JCPDS standards (ref. code: 00-004-0777, CaO). We can also notice the effect of annealing temperature on the structural parameters of the NdVO_4_ crystallites. An increase in the values of the 2θ peaks for NdVO_4_ is observed. In this case, we hypothesise that this phenomenon could be caused by a partial isomorphic replacement of neodymium with calcium (difference in ionic radii: Nd = 229 pm, Ca = 231 pm) or removal of various types of defects and impurities from the neodymium crystal structure in the process of thermal treatment. Explanation of this phenomenon requires more precise investigation using XRD analysis. The diatom biomass pyrolysis at 800 °C resulted in the complete decomposition of calcite CaCO_3_ and calcium oxide formation [27]. 

The results of thermogravimetric analysis (TG, thermogravimetric analysis (green line); DTG, thermogravimetric derivative analysis (purple line); and DSC, differential scanning calorimetry (red line)) performed for NdVO_4_-doped diatom biomass (SiO_2_/CaCO_3_/C_org_/NdVO_4_NPs) are presented in Figure 4. The thermogravimetric curve shows three different stages of weight loss, which connects with phase transformations in the synthesised composite. The first stage, with a mass loss of about 12%, appeared in the temperature range of 111–199 °C. This mass loss is associated with a clear peak on the DTG curve and is attributed to dehydration processes. The second stage occurred in the temperature range of 394–529 °C with a mass reduction of about 19%. It was characterised by an exothermic effect visible on the DTA curve and an intense DTG peak centred at 500 °C. This stage is associated with diatom organic matter degradation. The third stage appears in the temperature range 602–764 °C with a mass loss of nearly 30%, which is affected by an asymmetric DTG peak and related to the calcite CaCO_3_ decomposition effect on the DTA curve with carbon dioxide emission [29]. 

**Figure 5:**
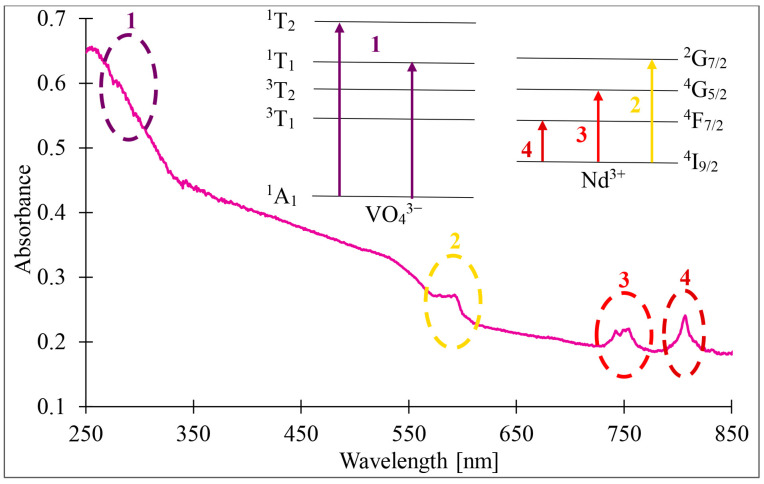
UV-vis absorption spectrum of pyrolysed diatom biomass doped with NdVO_4_ with schematic diagram of energy levels responsible for different peaks (inset).

The UV-vis absorption spectrum of pyrolysed diatom biomass doped with NdVO_4_ is shown in Figure 5, covering the range of 250 nm to 850 nm. The spectrum exhibits four distinct absorption peaks at 297 nm, 593 nm, 753 nm, and 817 nm, indicating absorption of both UV and visible light. The peak at 297 nm is most likely due to an electron transition in VO_4_^3−^, corresponding to the transition of electrons from the unbound O 2p states to the V 3d and anti-bound O 2p states [30,31]. The absorption peaks at 593 nm, 753 nm, and 817 nm are primarily due to the electron transition of Nd^3+^, specifically the 4f transitions from ^4^I_9/2_ to ^2^G_7/2_, ^4^G_5/2_, and ^4^F_7/2_ [32,33]. This is illustrated in the inset in Figure 5.

**Figure 6:**
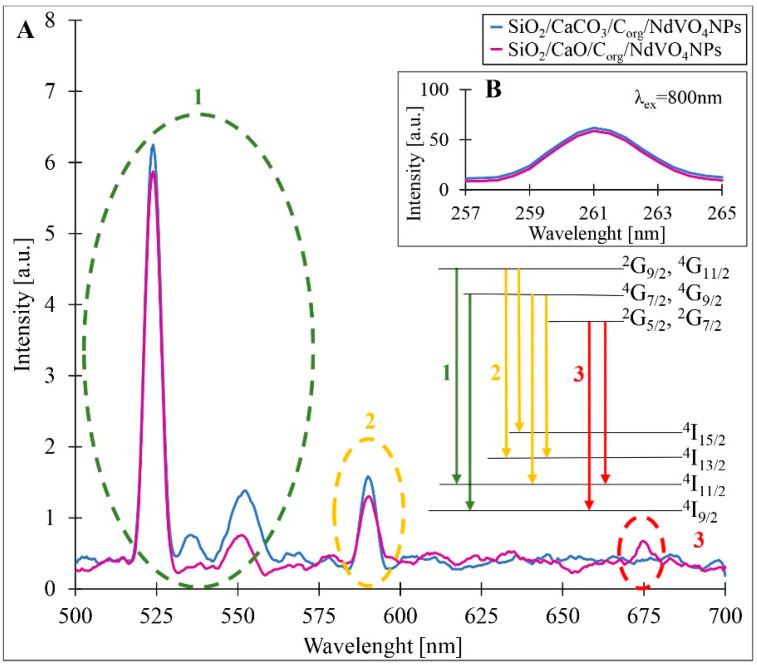
Upconversion luminescence spectra of the obtained composites at the excitation wavelength of 800 nm for the range of 500–700 nm (**A**) and the range of 257–265 nm (**B**). Schematic diagram of the energy levels responsible for the various peaks (inset).

The study of the photoluminescence properties of the obtained composites showed that they possessed high emission intensities in ultraviolet (376 nm), green (535 nm), orange (635 nm), and red (677 nm) light, regardless of the excitation wavelength used. When excited in the near-infrared (λ_ex_ = 800 nm), the resulting composites show four anti-Stokes emission bands in the visible region, as shown in the emission spectrum in Figure 6. The emission in the ultraviolet region (~261 nm) is most likely the result of an electronic transition in VO_4_^3−^, which corresponds to the transition of electrons from the V 3d and O 2p bonding states to the non-bonding states [9,34]. Green, orange, and red light emissions are mainly due to electron transitions in Nd^3+^ [28]. The strong green emission band (~523 nm) can be attributed to the transitions (^2^G_9/2–_^4^G_11/2_) → ^4^I_11/2_ and (^4^G_7/2–_^4^G_9/2_) → ^4^I_9/2_, while the orange emission (~590 nm) can be assigned to transitions (^2^G_9/2–_^4^G_11/2_) → ^4^I_13/2_, ^4^I _15/2_ and (^4^G_7/2_- ^4^G_9/2_) → ^4^I_11/2_, ^4^I _13/2_. Emission in the red light range (~675 nm) may be caused by transitions (^4^G_5/2–_^2^G_7/2_) →, ^4^I_9/2_, ^4^I_11/2_.

It can also be noted that the upconversion luminescence was obtained for synthesis composite only with xenon lump but not with lasers yielding very high excitation photon densities. Earlier, the several well-resolved narrow bands in the 660 nm–760 nm spectral range were obtained under CW excitation at 785 nm for Nd^3+^ doped yttrium orthoaluminate nano-perovskites (Nd^3+^:YAlO_3_) [35]. The near-infrared anti-Stokes luminescence at 740 nm and 800 nm exhibited under the excitation at 980 nm was observed for perovskite calcium titanate particles CaTiO_3_ co-doped with Yb^3+^ and Nd^3+^ ions [36]. Recently, Singh and co-workers [37] reported the luminescence with anti-Stokes emissions in the visible region at 489 nm, 540 nm, 605 nm and 671 nm under 806 nm excitation of the Nd_2_O_3_ doped borophosphate glasses.

The fluorescence quantum yield $\left(n_{{fl}_{sample}}\right)$ of a solution containing SiO_2_/CaO/C_org_/NdVO_4_NPs in 96% ethanol was determined using a relative method. The concentration of the analysed sample was 0.027 mg/mL. The data required for calculating $n_{{fl}_{sample}}$ can be found in Table 1. 

The luminescence quantum yield was calculated using Equation (6), resulting in a value of 0.0013 (0.13%). The luminescence quantum yield of neodymium-doped materials highly depends on the concentration of Nd^3+^ in the sample [38]. In Nd^3+^ doped systems, the presence of cross-relaxation and energy migration processes between Nd^3+^ ions cause the quantum yield to decrease as the concentration of Nd^3+^ increases [39]. There is an optimal concentration of neodymium that maximises luminescence brightness. However, accurately determining this concentration requires an absolute determination of n_fl_.

It should also be noted that the obtained composite of pyrolysed diatom biomass doped with neodymium orthovanadate nanoparticles, in addition to the detected luminescent properties (upconversion luminescence, narrow-band, and significant shifts of anti-Stokes emission), is characterised as biocompatible, non-toxic, thermally, and chemically stable materials. The materials with such properties are in demand for applications in phototherapy, bioimaging and biosensing [12], or in solar spectrum conversion [40].

## 4. Conclusions

This study demonstrates the feasibility of synthesizing SiO_2_/CaO/C_org_/NdVO_4_NPs or SiO_2_/CaCO_3_/C_org_/NdVO_4_NPs composites with anti-Stokes photoluminescence using the green method of the metabolic doping of diatom cells by neodymium and vanadium during the process of diatoms growing. The synthesis of the crystalline NdVO_4_ nanoparticles is performed using unicellular microalgae (diatoms) in its eco-friendly growth medium at room temperature in an ambient atmosphere. It was established that diatom cells (pyrolysed and unpyrolysed) are covered by crystallite clusters of neodymium orthovanadate nanoparticles in the form of single-layer irregular flakes. We believe that this work gives a new approach to developing novel green methods of NdVO_4_ nanoparticle synthesis and opens up the possibilities to obtain new REE-added composite materials with specific upconversion luminescence.

## Figures and Tables

**Table 1:** Summary of results required to calculate fluorescence quantum yield using the relative method.

$n_{{fl}_{reference}}$	$n_{{fl}_{sample}}$	${IF}_{sample}$	${IF}_{reference}$	$n_{sample}$	$n_{reference}$	$n_{sample}^{2}$	$n_{reference}^{2}$
0.59	0.0013	16.58	7658.98	1.364	1.346	1.859	1.812

## Data Availability

Data will be made available on request.
